# Treatment of primary pterygium: Role of limbal stem cells and conjunctival autograft transplantation

**DOI:** 10.4103/0974-620X.48418

**Published:** 2009

**Authors:** Mohamed A. E. Soliman Mahdy, Jagdish Bhatia

**Affiliations:** 1 Al-Hussein University Hospital, Al-Azhar University, Cairo-Egypt; 2 Department of Ophthalmology, Rustaq Hospital, Rustaq, Sultanate of Oman

**Keywords:** Pterygium, autograft, excision, stem cells

## Abstract

**Purpose:**

The limbus and its stem cells are very important in the pathogenesis of pterygium. In this study, the efficacy of limbal stem cells and conjunctival autograft transplantation for the treatment of primary pterygium will be assessed.

**Design:**

Prospective noncomparative cohort study.

**Materials and Methods:**

Forty-two eyes of 42 patients with grade I-III primary pterygium were included in the study. Pterygium excision was performed followed by superotemporal limbal stem cells and conjunctival autograft transplantation in all cases. Recurrence of pterygium and complications within a mean follow-up period of 18.26 months (10-28 months) was studied.

**Results:**

Recurrence of pterygium occurred in two eyes (2/42; 4.75%). No significant complications were noted. Apart from re-operation in the two recurrent cases, no further surgical interventions were needed in any case.

**Conclusions:**

Limbal stem cells and conjunctival autograft transplantation is a safe and effective technique for the treatment of different grades of pterygium. It is very useful in preventing pterygium recurrence, which is a major problem in pterygium surgery.

Pterygium is a fibrovascular overgrowth of bulbar conjunctiva across the limbus onto the cornea. It is seen more frequently in certain populations, and its incidence varies greatly in different geographical zones. Pterygium is thought to be caused by increased exposure to light, dust, dryness, heat, and wind. Pterygium was seen twice as frequent among persons who worked outdoors but was only one fifth as likely among those who always used sunglasses outdoors.[1] In the Barbados eye studies, the incidence of pterygium was high in black population, for an average of 1.3% per year. Working outdoors increased the risk 1.5-fold, whereas having a darker skin complexion and using eyewear for either reading or distance substantially decreased the risk of developing pterygium.[2] Ultraviolet (UV) light is one of the major factors implicated in the pathogenesis of pterygium, although the mechanism by which UV light induces this disease remains elusive.[3,4]

Heparin-binding epidermal growth factor (HB-EGF), a potent growth factor capable of stimulating altered cell growth and anchorage independence, has been implicated in the pathogenesis of pterygia.[5]

Surgical treatment of pterygium is directed towards excision, prevention of recurrence, and restoration of ocular surface integrity. A myriad of techniques, some combined with others, have been described for achieving these goals. The main complication of these procedures has been the recurrence rate, which has been estimated as high as 30%-70%[6] Surgical techniques for pterygium include bare sclera excision, excision with simple conjunctival closure, excision with administration of antimetabolite adjuvants such as mitomycin C (MMC),[7] excision with conjunctival autograft[8,9] and excision followed by amniotic membrane transplantation.[10] Treatments such as radiation therapy, the use of antimetabolites agents have succeeded in diminishing the number of recurrences from between 5% and 12%[2,4] However, serious complications are associated with these methods of treatment, such as secondary glaucoma, cataracts, uveitis, corneal perforation, and scleral necrosis, resulting in perforation and secondary endophthalmitis.[11,12]

In 1985, Kenyon and collaborators introduced conjunctival autograft as a technique for the treatment of recurrent or advanced pterygium.[8] Although this surgical technique is more time-consuming, it has reduced the number of recurrences with the same efficacy as the previously described treatments without the risk of potentially serious complications. Survival curve analysis showed that there was a 50% chance that there would be a recurrence of pterygium within the first 120 days, and a 97% chance of recurrence within 12 months of its removal.[13] Recently, the importance of limbal stem cells in the pathogenesis of pterygia has been reported,[14] and authors have suggested that a healthy limbus acts as a barrier to conjunctival overgrowth.[15] Conceptually, one could possibly reduce the pterygium recurrence by including healthy limbus in the conjunctival autograft. Besides, moving a limited area of limbus stem cells may not be detrimental to the ocular surface.

## Materials and Methods

The study was approved by the Hospital Scientific Committee and written informed consent was taken from all patients included in the study. The participants were patients attending Rustaq General Hospital. Forty-two eyes of 42 patients with grade I-III primary pterygium (3 patients with pterygium grade-I, 25 patients’ grade-II and 14 patients grade-III) were included in the study. The pterygium was graded depending on the extent of corneal involvement as described in a previous study.[16] Table 1 shows the patients’ demographic characteristics. All patients underwent pterygium excision with conjunctival autograft from the same eye.

**Table 1:** Demographic details of study patients, preoperative characteristics and postoperativeresults

*Number of eyes (n)*	*42*
Age	43.4 years + 13 years (21–66 years)
Sex	
Male	30 (71%)
Female	12 (29%)
Grades of pterygium	
Grade I	3 eyes (7.14%)
Grade II	25 eyes (59.52%)
Grade III	15 eyes (33.33%)
Preoperative visual acuity	0.32
Postoperative visual acuity	0.63
Follow-up period	18.26 months + 5.7 months
	(10–28 months)

All the operations were performed using peribulbar anesthesia with 2% lidocaine and 0.5% bupivacaine. To improve exposure, 6/0 silk or 8/0 Vicryl traction sutures were placed in the episcleral-limbal area at 12 o’clock position. The pterygium was removed starting with superior conjunctival incision. The conjunctiva was then dissected to free the body of pterygium. Superficial keratectomy was done to clean the cornea at the area covered by the head of pterygium using a Beaver blade. Minimal cautery was used to control bleeding and the area of bare sclera was measured. A superotemporal limbal conjunctival autograft incorporating a small portion of limbal stem cells, and measuring approximately 0.5-1 mm larger than the recipient bed, was harvested from the same eye. The graft was dissected towards the cornea with a number 15 scalpel blade to include 0.5 mm of the superficial limbus. Dissection of the conjunctival autograft was commenced superotemporally from the fornix to the limbus. Care was taken to include minimal or no Tenon’s capsule. The graft was transferred to the recipient bed and secured with 10/0 or 8/0 Vicryl sutures. The graft was oriented so that harvested limbal stem cells were positioned adjacent to the cornea. The host area was left with Tenon’s capsule exposed. Postoperatively, the patients were treated with a tapering dose of topical dexamethasone and antibiotic drops (four times daily for 2 weeks, three times daily for 2 weeks, and two times daily for 2 weeks). The follow-up period ranged from 10 to 28 months.

## Results

**Figure 1:**
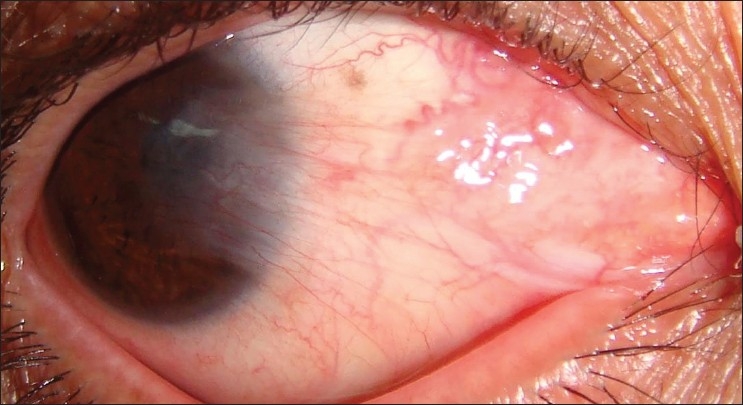
Preoperative photograph of an eye with pterygium

**Figure 2:**
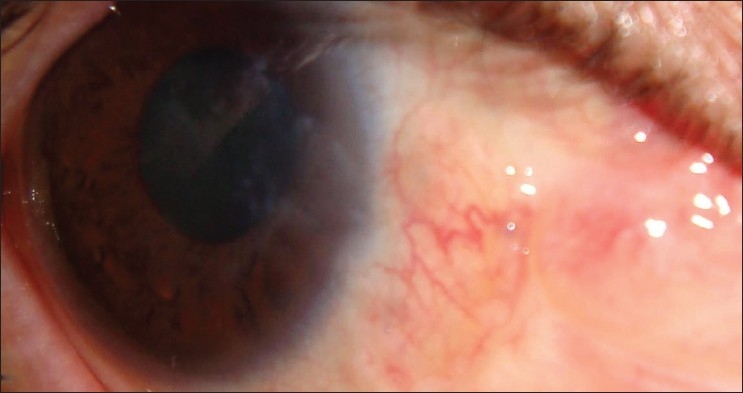
Postoperative photograph of the same eye 3 months after surgery

The demographic patients’ characteristics, preoperative and postoperative data of cases are shown in Table 1. During the follow-up period recurrence of pterygium beyond the limbal edge was noted in two eyes (4.75%). The sites from which the grafts were harvested, epithelialized completely without any significant scarring. No dehiscence, epithelial inclusion cysts or Tenon’s granulomas were observed. No symblepharon or ocular motility disturbances were reported in any case. The mean visual acuity improved from 0.32 to 0.63 (decimal notation). Figures 1 and 2 show a case before operation and 3 months after surgery.

## Discussion

The current study included 42 patients with pterygia surgically treated with conjunctival and limbal stem cells autograft. The results showed that after a period of follow up that varied from 10 to 28 months, 95.25% of cases were successfully treated without recurrence of pterygial growth or significant complications. Only 4.75% of cases showed recurrence of pterygial growth.

Varied surgical procedures have been described for the treatment of pterygium. The high postoperative recurrence rates indicate that there is still no single definitive treatment. The ideal surgical technique should be one that effectively prevents recurrences without development of complications. Of the procedures used most often to treat recurrent or advanced pterygium, the one that comes closest to achieving this goal is, probably, the conjunctival autograft described by Kenyon *et al*.[8] This procedure reduces recurrence with minimal complications when compared with the use of β-radiation or MMC. However, recurrences were not completely eliminated, especially in patients who live in areas with high levels of ultraviolet light.[17]

Considering the importance of the limbus and its stem cells in the pathogenesis of the pterygium, a new technique has been developed that includes in addition to the conjunctival autograft, a part of the limbal stem cells that aids in the complete anatomic and physiologic reconstruction of the excised pterygium area. This limbal reconstruction may theoretically reduce the recurrence rate. The complication rate, however, of this method should not be greater than that of the procedure described by Kenyon.[18] The surgical procedure used in the current study differs only in the fact that a small portion of limbus is included in the graft. Researchers who have used larger areas of limbal resection for grafts in the opposite eye have not reported higher complication rates.[19]

Complications related to conjunctival autograft transplantation include transient graft edema, corneoscleral dellen, graft retraction, epithelial cysts, and Tenon granuloma.[20] A more significant complication such as graft necrosis may occur if the graft is not properly oriented or placed on a completely avascular zone. None of these complications developed in our patients.

Shimazaki *et al*,[21] used a more or less similar technique as described by us to treat two groups of patients, 16 of them with primary pterygium. After an average follow-up period of 10.5 months, only 7.4% showed slight recurrence with 1 mm of extension beyond the limbus, and no need for additional surgery. The average age of these study patients was 61 years as compared to 41 years in our study population. Only two of the patients were less than 40 years of age. Gris *et al,* used a similar technique closer to the one used in our study in 7 patients with recurrent pterygium. They reported no recurrence or significant complications.[22] In another study, two groups of patients with pterygium who underwent excision were studied. One group underwent limbal conjunctival autograft with amniotic membrane transplant. This group showed no recurrence of pterygium. Another group underwent MMC and amniotic membrane transplant - this group had a recurrence rate of 20%[23] Minimal limbal conjunctival autograft has been used with a recurrent rate of 9.2% during a follow up period of 6-29 months.[24]

The recurrence rate of 4.75% reported in our study is one of the lowest reported so far and may be due to the use of a very thin conjunctival graft devoid of Tenon’s tissue in addition to incorporating a part of the adjacent limbal stem cells in the graft. If we considered the relatively young age of the patients operated and the geographic area in which these patients are living (windy, sandy with high and prolonged exposure to UV light, as they are living and working in the Arabian Gulf region), this rate of recurrence is even an excellent rate. Giving the above result, we conclude that despite the fact that limbal stem cells and conjunctival autograft transplantation is a time consuming procedure, it is safe and effective technique for the treatment of different grades of pterygium. It is very useful in prevention of pterygium recurrence, which is a major problem in pterygium surgery.
